# Role of tongue pressure production in oropharyngeal swallow biomechanics

**DOI:** 10.1002/phy2.167

**Published:** 2013-11-29

**Authors:** Kazuhiro Hori, Hiroshige Taniguchi, Hirokazu Hayashi, Jin Magara, Yoshitomo Minagi, Qiang Li, Takahiro Ono, Makoto Inoue

**Affiliations:** 1 Division of Dysphagia Rehabilitation, Niigata University Graduate School of Medical and Dental SciencesNiigata, Japan; 2 Department of Prosthodontics, Gerodontology and Oral Rehabilitation, Osaka University Graduate School of DentistrySuita, Japan; 3 Department of General Dentistry & Emergency, School of Stomatology, The Fourth Military Medical UniversityXi'an, China

**Keywords:** hyoid movement, Swallowing, tongue, tongue pressure, videofluorography

## Abstract

The tongue is important for orofacial movements, including swallowing. Although numerous studies have focused on tongue pressure against the palate, its physiological role has not been fully evaluated. The tongue pressure generation may have the temporal coordination with the swallowing relational organs. The aim of this study was to clarify the physiological mechanisms of tongue pressure and to investigate the temporal relationship among tongue pressure, supra-hyoid muscle activity, and videofluorographic (VF) images during swallowing. Fifteen healthy young subjects participated. Tongue pressure measured using a sensor sheet with five channels, electromyographic EMG, and VF was recorded synchronously during 4-ml barium swallowing. Swallowing behavior in VF images with and without the sensor sheet was compared. Furthermore, the temporal relationship between events measured from tongue pressure, EMG, and VF was evaluated. Swallowing behavior on VF images was not affected by placement of the sensor sheet. Tongue pressure at the posterio-lateral point of the hard palate tended to have biphasic peaks. Tongue pressure production with a monophasic pattern appeared during the same period as the second peak in the biphasic pattern. The onset of tongue pressure was later than the start of hyoid movement and onset of EMG, and offset was observed between the hyoid at the up-forward position and reposition. Onset of tongue pressure at the anterior area was correlated with the start of slight hyoid elevation. Offset of tongue pressure at the posterio-lateral points was strongly time locked with the hyoid at the up-forward position. The present results suggested the temporal coordination of tongue pressure generation with the swallowing-related organs. That is, the tongue pressure was produced for bolus propulsion, and was closely related to hyoid movement temporally during swallowing. These results may contribute to clarify the clinical state with the disorder of tongue kinetics.

## Introduction

### Dysphagia

Dysphagia, a disorder of the feeding mechanism in humans, has become a matter of increasing concern in recent years, particularly as a result of global aging. In dysphagic patients, motor disorder of the tongue and incoordination of tongue and jaw movement are frequently seen, and this influences the oral and pharyngeal phases of swallowing. The absence of adequate protocols or devices was believed to be responsible for the lack of attention on tongue activity in the evaluation of dysphagia, despite its importance in whole sequence of feeding function. Videofluorography (VF) is still recognized as the “gold standard” in clinical practice. VF can simultaneously obtain the movement of food bolus and swallowing-related organs (Palmer et al. 1992). Although VF investigations have qualitatively revealed the coordination of tongue and jaw movement in mastication and swallowing, VF images do not provide quantitative biomechanical information and it is difficult to apply VF widely and repeatedly because of radiation exposure (Wright et al. 1998).

### Tongue pressure

Because the tongue plays an important role by contacting the palate during swallowing, numerous investigations on contact between the tongue and hard palate during swallowing have been performed (Shaker et al. 1988; Chiba et al. 2003; Ono et al. 2004; Hori et al. 2006; Kieser et al. 2008; Kennedy et al. 2010), and these have used pressure sensors to measure the magnitude of tongue pressure. Since the 1990s, the biomechanical significance and age- and gender-related differences in tongue pressure production have been investigated in several studies using intraoral sensing probes (Ono et al. 2010). Intraoral sensing probes such as the Iowa Oral Performance Instrument (IOPI) and handy probe (Hayashi et al. 2002; Utanohara et al. 2008) are useful for evaluating the maximal isometric contraction of the tongue. Although some researchers (Youmans and Stierwalt 2006; Youmans et al. 2009; Steele et al. 2012) have attempted to apply this type of device to the evaluation of swallowing kinetics, insertion of a balloon with a certain volume and resistance into the oral cavity and interference of the cable with mouth closing prevents evaluation of physiologically natural swallowing kinetics. In addition, the standardization of measurement points has been insufficient because of the hand-held probe design. Kieser et al. (2008) measured the buccal and tongue pressure using an experimental plate installed eight pressure sensors. In addition, they reported that there were significant negative pressures in the mouth during swallowing, and that pressure profiles varied markedly between individuals. We also described the normal pattern of tongue pressure production in voluntarily evoked swallowing (Ono et al. 2004) and the chewing of gummy jelly (Hori et al. 2006) using an experimental palatal plate with seven pressure sensors.

Although the experimental palatal plate was able to accurately measure tongue pressure at multisensory points during natural swallowing, construction of the device was so complex and inefficient that it was unsuitable for clinical use. We therefore developed a novel sensor sheet for measuring tongue pressure as a simple and mobile procedure to evaluate tongue function in mastication and swallowing (Hori et al. 2009). This system for measuring tongue pressure has been supplied as a ready-to-use product, easily adhering to the hard palate during use.

The sensor sheet is only 0.1 mm thick and has five sensing points. The pathway of the cable of the sensor sheet was designed to avoid interfering with physiological swallowing through occlusal contact. These advantages were also considered to be quite effective for reducing discomfort in the oral cavity. Using this system, tongue pressure in elderly people (Tamine et al. 2010) and stroke patients (Hirota et al. 2010) were measured, and this was found to be useful for quantitative evaluation of tongue activity in dysphagic patients.

Despite of the development of such equipment, the effects of the apparatus insertion into the oral cavity on swallowing behavior have not been assessed.

### Wave of tongue pressure

In this series of research into tongue pressure measurement, we noted that the tongue pressure in healthy subjects showed monophasic or biphasic patterns (Hori et al. 2009). Taniguchi et al. (2008) reported that the tongue pressure wave measured with sheet-type sensors had single peak. On the other hand, Kennedy et al. (2010) found that the tongue pressure using an experimental plate with pressure sensors showed a biphasic pattern. However, few researchers have focused on the differences in monophasic and biphasic patterns. It has not yet been clarified whether the number of phases of tongue pressure changed accidentally through movement of the tongue, or whether it was due to an adaptive phenomenon during swallowing. Therefore, the features of these patterns of tongue pressure should be investigated.

### Tongue pressure with another device

Although numerous previous studies focused on the measurement of tongue pressure against the hard palate to evaluate swallowing function, few studies have focused on the physiological role of the pressure in food processing and propelling. Tongue pressure represents the contact force between tongue and hard palate; however, movement of the bolus or oropharyngeal organs cannot be identified by tongue pressure. It has not been sufficiently clarified on the tongue pressure wave, and only tongue pressure measurements have limitations in explaining swallowing behavior. Thus, tongue pressure measurement should be performed with other bio-functional parameters. Several reports have attempted to evaluate tongue pressure and other devices in order to explain the relationship between tongue pressure and oropharyngeal organs. Taniguchi et al. (2008) and Ono et al. (2009) reported that the onset of electromyographic (EMG) activity of supra-hyoid preceded the onset of tongue pressure. They also noted that the peak of tongue pressure at the anterior or posterior area had a relationship with the tail of the bolus at the fauces. Despite this approach, few reports have succeeded in explaining the relationship between tongue pressure production and the kinetics of swallowing-related organs.

### Purpose

Knowledge on tongue pressure measurement is not only based on the amplitude of tongue force; the duration and characteristic wave patterns of tongue pressure are also valuable information. Moreover, it should be considered how the insertion of a sensor sheet for tongue pressure measurement affects the swallowing behavior.

In this study, therefore, we performed a comparison of bolus transport and movement of related organs in VF images with and without the sensor sheet for measuring tongue pressure in order to assess the effects of placement of the sensor sheet on the swallowing behaviors. Next, we synchronously recorded tongue pressure, EMG activity of supra-hyoid muscles, and VF images to compare the properties of monophasic and biphasic tongue pressure pattern and to investigate the temporal relationship among the generation of tongue pressure, the activity of supra-hyoid muscles, and the movement of bolus and hyoid bone during swallowing.

## Material and Methods

### Subjects

Fifteen healthy subjects (11 men and 4 women; age range, 24–31 year; mean, 27.3 ± 2.5 years) without disturbance of mastication and deglutition, abnormality in the number or position of teeth except for the third molar, history of orthodontic treatment and temporomandibular disorders, and abnormality in occlusion were included in this study. Written informed consent was obtained from each subject after explaining the aim and methodology of the study. This study received approval by the ethical committee of Niigata University Faculty of Dentistry.

### Equipment

#### Tongue pressure

**Figure 1:**
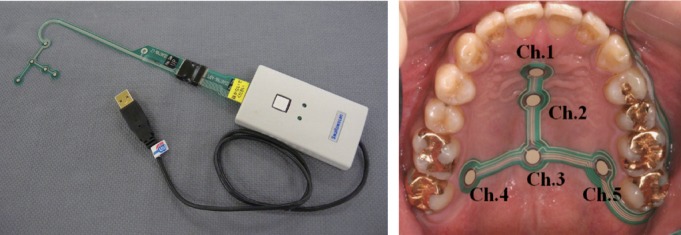
Swallow scan system and location of sensing points. Left: swallow scan system and sensor sheet. Right: intraoral view of attached sensor sheet and location of sensing points.

The tactile sensor system Swallow Scan (Nitta, Tokyo, Japan) with a special sensor sheet for measuring tongue pressure was used in this study (Fig. 1) (Hori et al. 2009). The thickness of sensor sheet is about 0.1 mm and it has five measuring points. Three measuring points (Chs 1–3) were placed along the median line (Ch 1 was set at the anterior-median region, Ch 2 was set at the mid-median region, Ch 3 was set at the posterior-median region), and two sensors (Chs 4 and 5) were situated in the posterior-circumferential regions of the hard palate. A small, medium, or large sensor sheet was selected for each subject according to the size of the hard palate. Before recording, the sensor sheet was attached to the palatal surface of the palatal mucosa directory with a sheet-type denture adhesive (Touch Correct II; Shionogi, Osaka, Japan). The wire was then connected to the computer exiting the oral cavity via the oral vestibule to avoid interference with the occlusion. After attaching the sensor sheet to the palate, a calibration was performed by applying negative pressure on the cable of sensor sheet using a vacuum pump. The pressure measured by the sensors is thus transmitted in real time to a personal computer, in which the data are displayed and saved at 100 Hz.

#### Videofluorography

The oral and pharyngeal organs of the subjects and bolus movement were observed using VF (ULTIMAX 80, Toshiba Co., Tokyo, Japan). VF images were acquired in the sagittal plane. VF images at a speed of 30 frames per second were converted and recorded to another computer through the AD board (Power Lab ML880; AD Instruments, Colorado Springs, CO). The total exposure to radiation per session was estimated to be 89.77 mGy and was limited to a maximum of 2 min per subject.

#### Electromyography

Pairs of surface electrodes with a diameter of 8 mm (NT-211u or NT-212u; Nihon Kohden, Tokyo, Japan) were used for EMG recordings of both sides of the supra-hyoid muscle group. Two electrodes were attached to the skin over the anterior belly of the digastric muscle with an interelectrode distance of 2 cm. A reference electrode was affixed to the earlobe.

Signals from the EMG electrodes were amplified (Dual Bio Amp; AD Instruments) and stored on a computer through an interface at 10 kHz.

#### Synchronizing system

Tongue pressure, EMG activity, and VF images during swallowing were recorded synchronously. To synchronize all data, the synchronizing signal from Swallow Scan was recorded to all computers.

### Data collection

In order to compare the swallowing behavior with/without the sensor sheet for tongue pressure, VF images during liquid swallowing were recorded for six male subjects (27.7 ± 1.4 years). Subjects were asked to sit on a chair with their head vertical to the Frankfort plane. After 4 mL of barium liquid (40% wt/volume%) was inserted into the mouth by the experimenter, the subject kept it on the floor of the mouth, and then swallowed it on cue in a single swallow (dipper-type swallow) (Dodds et al. 1989). The order of measurements with and without the sensor sheet was randomized and measurements were performed three times each.

Next, synchronized measurements for VF, tongue pressure, and EMG in all fifteen subjects (11 males, four females, 27.3 ± 2.5 years) were performed. Posture and instructions for swallowing were the same as described above. Synchronous data on tongue pressure, EMG, and VF images during 4-mL barium liquid swallowing (dipper-type swallow) were recorded. Measurement was performed three times for each subject. We collected data from 45 swallows.

### Data analysis

Data analysis was performed using the Power Lab software package (Lab Chart 6 for Windows; AD Instruments).

### Swallowing events on VF images

VF images were analyzed using single-frame analysis (Taniguchi et al. 2008). The times of each event for hyoid movement, bolus movement, and velo-pharyngeal closure were determined by directly reading the digital clock on each video frame. Using this method, the times of the variables in Table 1 were determined.

**Table 1:** Analysis items on VF images

	Time event	Defined as
A	Start of slight hyoid elevation	Onset of slight hyoid elevation before swallowing reflex
B	Start of rapid hyoid elevation	Onset of rapid hyoid elevation for swallowing reflex
C	Hyoid reaches most up-forward position	Hyoid reaches most up-forward position
D	Hyoid bone repositioning	Hyoid repositions after swallowing
E	Tip of tongue touches palate	Onset of propulsive tongue tip movement, representing the tongue tip touching the palate
F	Tip of bolus reaches fauces	Passage of bolus head through the fauces
G	Tail of bolus passes fauces	Passage of bolus tail through the fauces
H	Tip of bolus reaches UES	Bolus head reaches the pharyngo-esophageal junction, which is known as the region of the UES
I	Tail of bolus passes UES	Passage of bolus tail through the UES
J	Onset of velopharyngeal closure	Onset of velopharyngeal closure
K	Offset of velopharyngeal closure	Offset of velopharyngeal closure

	Duration	Defined as
	Hyoid elevation time	From B. to C.
	Hyoid declination time	From C. to D.
	Hyoid movement time	From B. to D.
	Total swallowing time	From E. to I.
	Oral transit time	From E. to F.
	Oral clearance time	From E. to G.
	Pharyngeal transit time of bolus head	From F. to H.
	Pharyngeal transit time of bolus tail	From G. to I.
	Pharyngeal clearance time	From F. to I.
	Velo-pharyngeal closure time	From J. to K.

VF, videofluorography; UES, upper esophageal sphincter.

Subsequently, durations related to hyoid movement, bolus movement, and velo-pharyngeal closure were also calculated. Calculated durations are also shown in Table 1.

After the definition of time events and calculation of durations, the durations for hyoid, bolus, and soft palate movements during swallowing with and without the sensor sheet were compared. Moreover, the temporal relationships of all events from VF, EMG, and tongue pressure were evaluated. Onset time of rapid hyoid elevation was set as a reference time.

### Tongue pressure

**Figure 2:**
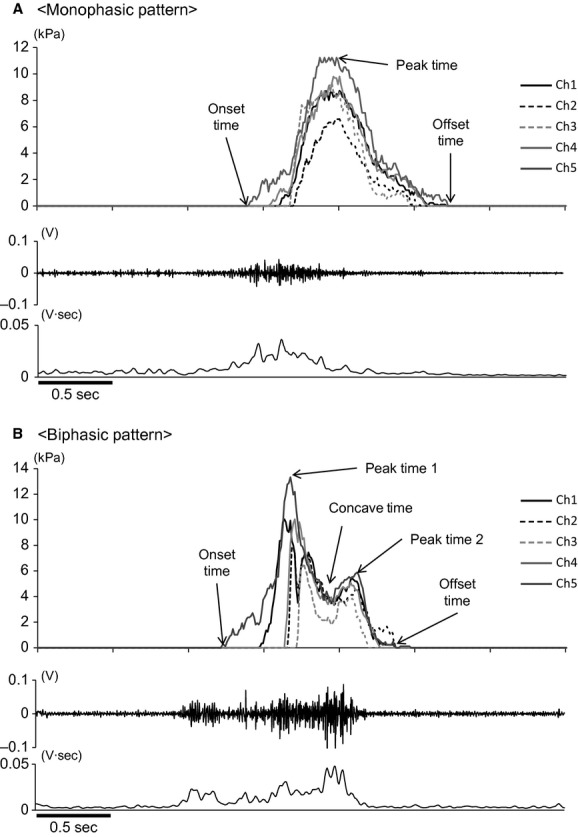
Sample of tongue pressure and EMG with monophasic and biphasic peak pattern. (A) Monophasic pattern. (B) Biphasic pattern. EMG, electromyography.

There were two patterns of tongue pressure waveforms; monophasic peaks and biphasic peaks (Fig. 2). The onset, peak, and offset times were evaluated from tongue pressure waveforms. In the case of biphasic wave, the onset, first peak, concave, second peak, and offset times were evaluated. Temporal relationships between the events measured from the tongue pressure, EMG, and VF recordings were evaluated. The maximal amplitude, duration, and area of tongue pressure for every channel were also obtained.

### EMG activity

EMG bursts were full-wave rectified and smoothed (time constant, 20 msec, Fig. 2). In a single burst, the times of onset, peak, and offset were obtained. Subsequently, the time sequence was calculated using the onset of hyoid elevation as a reference. Onset time of EMG burst was set as the time beyond 2 standard deviations (SD) of baseline validation, and offset time was when it was less than 2 SD.

### Statistical analysis

For comparison of duration on VF images with and without the sensor sheet, Student's *t*-test was performed. One-way analyses of variance (ANOVA) and Tukey's post hoc test were employed to examine the time sequences of VF images, muscle activity, and tongue pressure, and the differences in time sequences of monophasic or biphasic tongue pressure patterns. To examine the relationships between each time sequences, interclass correlation coefficients were calculated. Statistical analysis was performed using SPSS 16.0J (IBM Japan, Tokyo, Japan) and statistical significance was set at *P* < 0.05.

## Results

Because four VF images were not sufficiently clear for analysis, 41 swallows were analyzed.

### Comparison of swallowing behavior in VF images with/without sensor sheet

**Figure 3:**
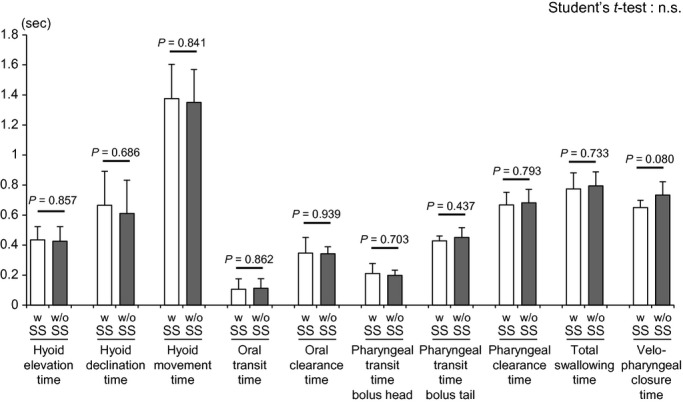
Comparison of time duration with/without sensor sheet in VF images. Values are means ± SD and *P* value (Student's *t*-test). Durations with/without sensor sheet showed no significant differences.

Figure 3 shows the duration of hyoid, bolus, and soft palate movement in VF images from six subjects. Durations of hyoid, bolus, and soft palate movement showed no significant differences, irrespective of whether sensor sheets for tongue pressure were present (*P* > 0.05).

### Waveform of tongue pressure

There were two patterns of tongue pressure waveforms; monophasic peaks and biphasic peaks (Fig. 2). In most swallowing cases, Chs 4 and 5 in the lateral posterior region showed biphasic peaks (Ch 4: 68.3%; Ch 5: 63.4%), and Chs 1 and 2 in the mid-anterior region also tended to show biphasic patterns (Ch 1: 58.5%; Ch 2: 58.5%). On the other hand, Ch 3 tended to show monophasic peaks, with the biphasic pattern rarely observed (Ch 3: 19.5%).

### Time sequence for tongue pressure in monophasic and biphasic patterns

**Figure 4:**
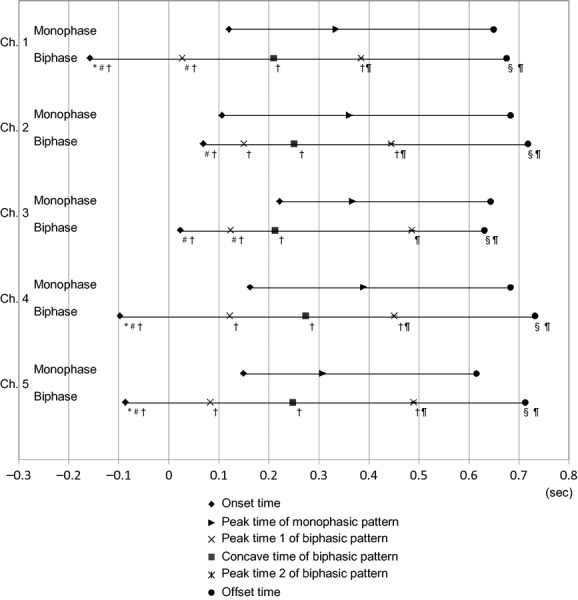
Comparison of time sequences for tongue pressure with mono- and biphasic patterns during 4-mL barium swallowing. Time “0” was set at the onset of rapid hyoid elevation. *The time event of biphasic pattern was significantly earlier than the onset time of monophasic pattern. ^#^The time event of biphasic pattern was significantly earlier than the peak time of monophasic pattern. ^†^The time event of biphasic pattern was significantly earlier than the offset time of monophasic pattern. ^§^The time event of biphasic pattern was significantly later than the onset time of monophasic pattern. ^¶^The time event of biphasic pattern was significantly later than the peak time of monophasic pattern. (ANOVA, Tukey's post hoc, *P* < 0.05). ANOVA, analyses of variance.

When the state of tongue pressure production was compared between monophasic and biphasic patterns in the time series where the onset of rapid hyoid elevation was set at 0 sec, the tongue pressure wave in the monophasic pattern existed around the second wave of the biphasic pattern. Onset in Chs 1, 4, and 5 with the biphasic pattern was significantly earlier than that with the monophasic pattern (Ch 1; *P* = 0.005, Ch 4; *P* = 0.001, Ch 5; *P* = 0.005). The onset time with the monophasic pattern did not have significant differences with the concave time of biphasic patterns (Ch 1; *P* = 0.929, Ch 2; *P* = 0.207, Ch 3; *P* = 1.000, Ch 4; *P* = 0.589, Ch 5; *P* = 0.758), and the peak time with the monophasic pattern did not show significant differences with the second peak time with the biphasic pattern (Ch 1, *P* = 0.879; Ch 2, *P* = 0.847; Ch 3, *P* = 0.651; Ch 4, *P* = 0.583; Ch 5, *P* = 0.319). This tendency was recognized on all channels. On the other hand, tongue pressure disappeared at almost the same time in both patterns on all channels (*P* > 0.05) (Fig. 4).

### Maximum amplitude, duration, and area of tongue pressure in monophasic and biphasic patterns

**Figure 5:**
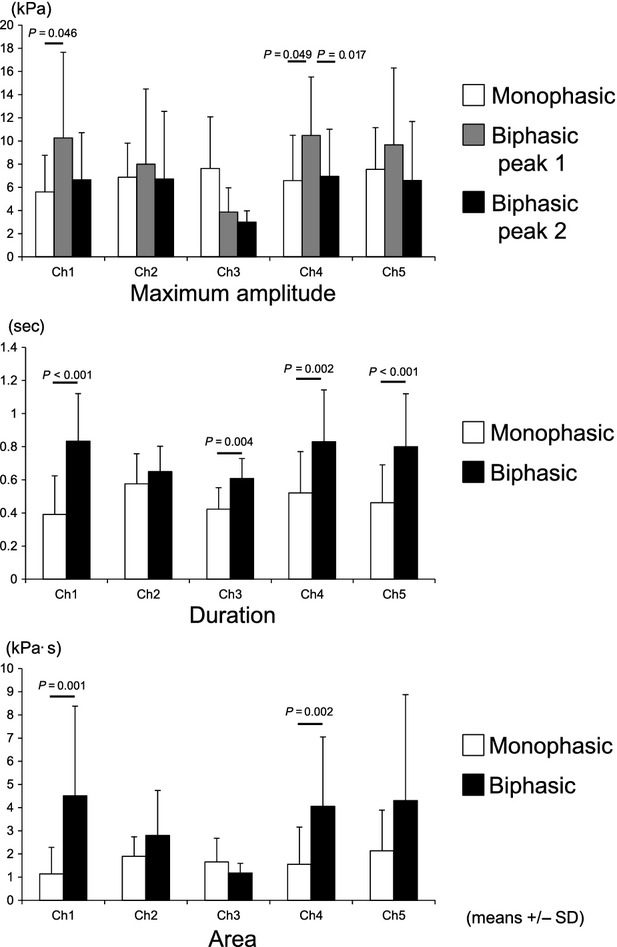
Maximum amplitude, duration, and area of tongue pressure. Values are means ± SD. Maximum amplitude of the first peak of the biphasic pattern at Ch 1 was larger than that of the monophasic pattern, and that of the first peak of the biphasic pattern at Ch 4 was larger than that of the monophasic pattern and the second peak of the biphasic pattern. Duration of the monophasic pattern was shorter than that of the biphasic pattern, except for Ch 2. The area with the monophasic pattern at Chs 1 and 4 was smaller than that with the biphasic pattern.

The maximum amplitude of tongue pressure in the monophasic pattern was almost the same as the second peak in the biphasic pattern. And it tended to be smaller than the first peak in the biphasic pattern at Chs 1 and 4. The duration in the monophasic pattern was shorter than that with the biphasic pattern in each channel, except at Ch 2. The area with the monophasic pattern on Chs 1 and 4 was smaller than that with the biphasic pattern (Fig. 5).

### Time sequence for tongue pressure, muscle activity, and VF images

**Figure 6:**
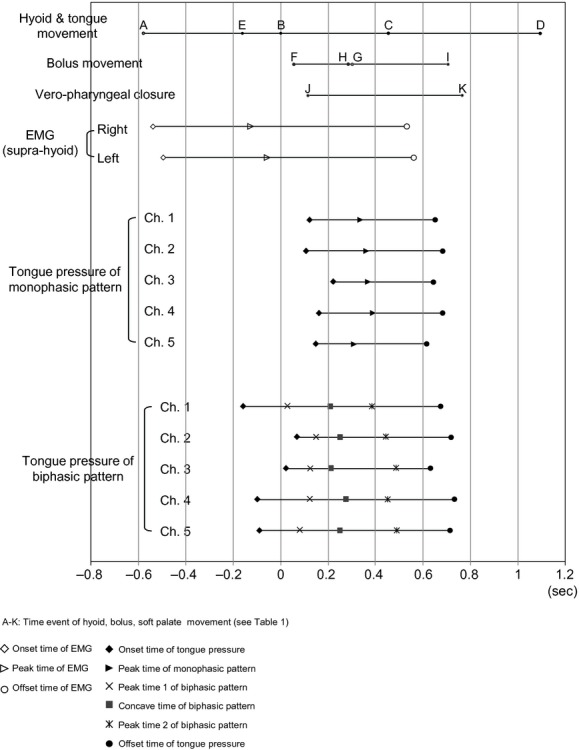
Time sequences during 4-mL barium swallowing. Time “0” was set at the onset of rapid hyoid elevation. (A–K) Time event of hyoid, bolus, soft palate movement (see Table 1).

Figure 6 and Table 2 show the time sequences for tongue pressure, muscle activity, and VF images during 4-mL barium liquid swallowing. The slight movement of the hyoid and onset of supra-hyoid muscle activity were observed initially, and the hyoid returned to its original position finally. The generation of tongue pressure (onset, peak time, and offset) was seen in these periods. The onset of slight hyoid elevation was significantly earlier than the onset of tongue pressure (*P* < 0.001). The onset of tongue pressure of biphasic pattern (especially at the Chs 1, 4, and 5) existed at almost same time as the tip of tongue touches palate, and was earlier than the time when the bolus tail passed the fauces or the bolus head reached to the upper esophageal sphincter (UES). The time when the bolus tail passed the fauces and that when the bolus head reached the UES was almost same, and located around the onset of monophasic tongue pressure or the concave time of biphasic pattern. The offset time of tongue pressure was between the hyoid at the most up-forward position and hyoid repositioning. In addition, it was almost the same as the offset of velopharyngeal closure and the time when the bolus tail passed the UES.

**Table 2:** Comparison of time sequence between time event on VF and tongue pressure

	Start of slight hyoid elevation	Hyoid reaches most up-forward position	Hyoid bone repositioning	Tip of tongue touches palate	Tip of bolus reaches fauces	Tail of bolus passes fauces	Tip of bolus reaches UES	Tail of bolus passes UES	Onset of velopharyngeal closure	Offset of velopharyngeal closure
Monophasic pattern										
Onset										
Ch. 1	−(0.000)	+(0.000)	+(0.000)	−(0.009)	(1.000)	(0.747)	(0.911)	+(0.000)	(1.000)	+(0.000)
Ch. 2	−(0.000)	+(0.000)	+(0.000)	−(0.001)	(1.000)	(0.169)	(0.370)	+(0.000)	(1.000)	+(0.000)
Ch. 3	−(0.000)	+(0.000)	+(0.000)	−(0.000)	(0.151)	(1.000)	(1.000)	+(0.000)	(0.975)	+(0.000)
Ch. 4	−(0.000)	+(0.001)	+(0.000)	−(0.000)	(1.000)	(0.979)	(0.998)	+(0.000)	(1.000)	+(0.000)
Ch. 5	−(0.000)	+(0.000)	+(0.000)	−(0.000)	(1.000)	(0.817)	(0.958)	+(0.000)	(1.000)	+(0.000)
Peak time										
Ch. 1	−(0.000)	(0.773)	+(0.000)	−(0.000)	−(0.019)	(1.000)	(1.000)	+(0.000)	(0.439)	+(0.000)
Ch. 2	−(0.000)	(1.000)	+(0.000)	−(0.000)	−(0.000)	(1.000)	(1.000)	+(0.000)	−(0.005)	+(0.000)
Ch. 3	−(0.000)	(0.999)	+(0.000)	−(0.000)	−(0.000)	(1.000)	(1.000)	+(0.000)	−(0.000)	+(0.000)
Ch. 4	−(0.000)	(0.999)	+(0.000)	−(0.000)	−(0.002)	(1.000)	(1.000)	+(0.000)	(0.111)	+(0.000)
Ch. 5	−(0.000)	(1.000)	+(0.000)	−(0.000)	−(0.000)	(1.000)	(1.000)	+(0.000)	−(0.020)	+(0.000)
Offset										
Ch. 1	−(0.000)	(0.466)	+(0.000)	−(0.000)	−(0.000)	−(0.000)	−(0.000)	(1.000)	−(0.000)	(1.000)
Ch. 2	−(0.000)	−(0.021)	+(0.000)	−(0.000)	−(0.000)	−(0.000)	−(0.000)	(1.000)	−(0.000)	(1.000)
Ch. 3	−(0.000)	−(0.027)	+(0.000)	−(0.000)	−(0.000)	−(0.000)	−(0.000)	(1.000)	−(0.000)	(0.869)
Ch. 4	−(0.000)	(0.086)	+(0.000)	−(0.000)	−(0.000)	−(0.000)	−(0.000)	(1.000)	−(0.000)	(1.000)
Ch. 5	−(0.000)	(0.708)	+(0.000)	−(0.000)	−(0.000)	−(0.000)	−(0.000)	(1.000)	−(0.000)	(0.836)
Biphasic pattern										
Onset										
Ch. 1	−(0.000)	+(0.000)	+(0.000)	(1.000)	+(0.009)	+(0.000)	+(0.000)	+(0.000)	+(0.000)	+(0.000)
Ch. 2	−(0.000)	+(0.000)	+(0.000)	−(0.002)	(1.000)	+(0.002)	+(0.008)	+(0.000)	(1.000)	+(0.000)
Ch. 3	−(0.000)	+(0.000)	+(0.000)	(0.925)	(1.000)	(0.084)	(0.172)	+(0.000)	(1.000)	+(0.000)
Ch. 4	−(0.000)	+(0.000)	+(0.000)	(1.000)	(0.345)	+(0.000)	+(0.000)	+(0.000)	+(0.004)	+(0.000)
Ch. 5	−(0.000)	+(0.000)	+(0.000)	(1.000)	(0.631)	+(0.000)	+(0.000)	+(0.000)	+(0.021)	+(0.000)
Peak time 1										
Ch. 1	−(0.000)	+(0.000)	+(0.000)	(0.066)	(1.000)	+(0.000)	+(0.000)	+(0.000)	(1.000)	+(0.000)
Ch. 2	−(0.000)	+(0.000)	+(0.000)	−(0.000)	(0.999)	(0.487)	(0.785)	+(0.000)	(1.000)	+(0.000)
Ch. 3	−(0.000)	+(0.005)	+(0.000)	(0.063)	(1.000)	(0.953)	(0.991)	+(0.000)	(1.000)	+(0.000)
Ch. 4	−(0.000)	+(0.000)	+(0.000)	−(0.000)	(1.000)	(0.067)	(0.207)	+(0.000)	(1.000)	+(0.000)
Ch. 5	−(0.000)	+(0.000)	+(0.000)	−(0.000)	(1.000)	+(0.004)	+(0.020)	+(0.000)	(1.000)	+(0.000)
Concave										
Ch. 1	−(0.000)	+(0.001)	+(0.000)	−(0.000)	(0.534)	(1.000)	(1.000)	+(0.000)	(1.000)	+(0.000)
Ch. 2	−(0.000)	+(0.022)	+(0.000)	−(0.000)	−(0.037)	(1.000)	(1.000)	+(0.000)	(0.742)	+(0.000)
Ch. 3	−(0.000)	(0.833)	+(0.000)	−(0.022)	(1.000)	(1.000)	(1.000)	+(0.000)	(1.000)	+(0.000)
Ch. 4	−(0.000)	(0.059)	+(0.000)	−(0.000)	−(0.002)	(1.000)	(1.000)	+(0.000)	(0.245)	+(0.000)
Ch. 5	−(0.000)	+(0.016)	+(0.000)	−(0.000)	−(0.049)	(1.000)	(1.000)	+(0.000)	(0.798)	+(0.000)
Peak time 2										
Ch. 1	−(0.000)	−(1.000)	+(0.000)	−(0.000)	−(0.000)	(1.000)	(0.998)	+(0.000)	−(0.000)	+(0.000)
Ch. 2	−(0.000)	−(1.000)	+(0.000)	−(0.000)	−(0.000)	(0.655)	(0.353)	+(0.000)	−(0.000)	+(0.000)
Ch. 3	−(0.000)	−(1.000)	+(0.000)	−(0.000)	−(0.000)	(0.926)	(0.798)	+(0.583)	−(0.000)	(0.077)
Ch. 4	−(0.000)	−(1.000)	+(0.000)	−(0.000)	−(0.000)	(0.407)	(0.164)	+(0.000)	−(0.000)	+(0.000)
Ch. 5	−(0.000)	−(1.000)	+(0.000)	−(0.000)	−(0.000)	(0.059)	−(0.015)	+(0.005)	−(0.000)	+(0.000)
Offset										
Ch. 1	−(0.000)	−(0.005)	+(0.000)	−(0.000)	−(0.000)	−(0.000)	−(0.000)	(1.000)	−(0.000)	(1.000)
Ch. 2	−(0.000)	−(0.000)	+(0.000)	−(0.000)	−(0.000)	−(0.000)	−(0.000)	(1.000)	−(0.000)	(1.000)
Ch. 3	−(0.000)	(0.958)	+(0.000)	−(0.000)	−(0.000)	−(0.005)	−(0.002)	(1.000)	−(0.000)	(1.000)
Ch. 4	−(0.000)	−(0.000)	+(0.000)	−(0.000)	−(0.000)	−(0.000)	−(0.000)	(1.000)	−(0.000)	(1.000)
Ch. 5	−(0.000)	−(0.000)	+(0.000)	−(0.000)	−(0.000)	−(0.000)	−(0.000)	(1.000)	−(0.000)	(1.000)

−: indicated that the time event on VF was earlier than that of tongue pressure.

+: indicated that the time event on VF was later than that of tongue pressure.

*P* value was enclosed in parentheses (analyses of variance [ANOVA] and Tukey's post hoc test (*P* < 0.05, *F* value = 81.783). VF, videofluorography; UES, upper esophageal sphincter.

### Relationship between events on tongue pressure and VF images

**Figure 7:**
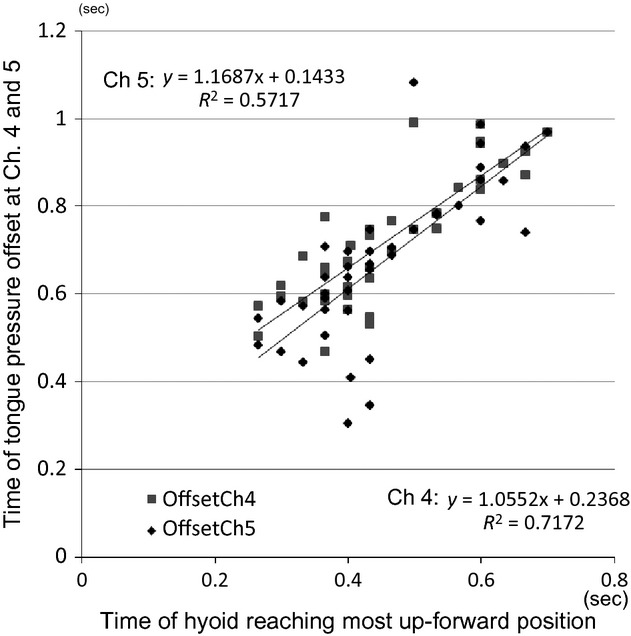
Relationship between time sequence of offset of tongue pressure at Chs 4 and 5, and time at which hyoid reached the most up-forward position on VF images. Offset time of tongue pressure at Chs 4 and 5 were correlated with hyoid at the most up-forward position. VF, videofluorography.

The interclass correlation coefficients between events of tongue pressure and movement of hyoid and bolus were calculated (Table 3). The onset of tongue pressure at Ch 1 at the mid-anterior region was temporally time-locked to the onset of slight hyoid elevation. The offset of tongue pressure at Chs 4 and 5 was strongly time locked with the hyoid reaching the most up-forward position (Fig. 7). On the other hand, hyoid repositioning had no significant correlation with tongue pressure. Timing of tongue tip contact with the palate on the VF images was correlated with onset on Chs 4 and 5, rather than on Ch 1. Time events for bolus movement, such as the tip or tail of bolus reaching or passing the fauces or UES, were strongly correlated with onset on Ch 3. Although the onset of soft palate elevation and velo-pharyngeal closure was also correlated with onset of Ch 3, the offset of velo-pharyngeal closure had no significant correlations with tongue pressure.

**Table 3:** Interclass correlation coefficients of time sequences between tongue pressure and VF images

	Tongue pressure														
	
	Onset					Peak time					Offset				
			
	Ch 1	Ch 2	Ch 3	Ch 4	Ch 5	Ch 1	Ch 2	Ch 3	Ch 4	Ch 5	Ch 1	Ch 2	Ch 3	Ch 4	Ch 5
Start of slight hyoid elevation	0.723	0.334	0.329	0.322	0.312	0.390	0.324	0.356	0.240	0.456	0.141	0.394	0.334	0.166	0.315
Hyoid reaches most up-forward position	0.210	0.567	0.678	0.130	0.178	0.237	0.440	0.508	0.202	0.212	0.634	0.496	0.528	0.827	0.690
Hyoid bone repositioning	−0.143	−0.087	0.020	0.264	0.293	0.168	0.134	0.117	0.347	0.255	0.107	0.102	0.228	0.101	−0.045
Tip of tongue touches palate	0.216	0.440	0.528	0.609	0.649	0.334	0.386	0.307	0.496	0.423	0.253	0.249	0.375	0.116	0.051
Tip of bolus reaches fauces	0.276	0.576	0.649	0.435	0.527	0.149	0.108	0.114	0.347	0.346	0.343	0.389	0.343	0.331	0.262
Tail of bolus passes fauces	0.334	0.541	0.604	0.005	0.076	0.202	0.476	0.463	0.057	−0.095	0.388	0.346	0.454	0.342	0.280
Tip of bolus reaches UES	0.351	0.559	0.627	0.374	0.469	0.251	0.257	0.312	0.238	0.390	0.417	0.489	0.517	0.436	0.352
Tail of bolus passes UES	0.154	0.471	0.589	0.306	0.423	0.085	0.125	0.082	0.079	0.196	0.275	0.283	0.237	0.255	0.139
Onset of velopharyngeal closure	0.154	0.418	0.629	0.276	0.379	0.100	0.195	0.339	0.204	0.301	0.234	0.371	0.491	0.509	0.321
Offset of velopharyngeal closure	0.153	0.345	0.296	0.144	0.286	0.223	0.097	0.107	0.350	0.268	0.400	0.322	0.220	0.359	0.219

UES, upper esophageal sphincter.

## Discussion

In this study, we measured tongue pressure using an original sensor sheet, and compared the swallowing behavior in VF images. We also attempted to define the function of tongue pressure within swallowing by recording muscle activity and assessing VF images. To our knowledge, this is the first report to investigate the temporal relationship among tongue pressure, activity of the supra-hyoid muscle, and movement of the hyoid and bolus.

Various equipments to determine tongue pressure have been developed. IOPI (Robbins et al. 1995; Crow and Ship 1996), which scans the air movement, is used most commonly. This device showed favorable performance in large studies because of its simplicity. However, it appears to be unsuitable for functional assessment, as it interferes with occlusion and has only one sensing point. Air-filled bulbs (Kay Pentax, Lincoln Park, NJ) uses the same principal, and has more than one sensing point. Some researchers (Hind et al. 2005; Ball et al. 2006; Steele and Huckabee 2007; Steele et al. 2010; van den Engel-Hoek et al. 2012) have used this device to measure tongue pressure during function, but it does not allow biting.

On the other hand, other investigations into the contact between the tongue and the hard palate have been performed with pressure sensors (Proffit 1972; Kieser et al. 2008; Kennedy et al. 2010) or transducers (Proffit et al. 1969). We also reported the normal pattern of tongue pressure production in voluntarily evoked swallowing using an experimental palatal plate with seven pressure sensors (Ono et al. 2004). However, this was too complex for clinical use, despite having the advantage of small size, and being capable of multiple sensing and physiological measurements.

The sensor sheet we developed has several advantages (Hori et al. 2009). It is a ready-made sensor sheet with five sensing points that are attached to the palate, and it requires no acclimation time. The design does not interfere with occlusion and allows physiological function. The development of this tongue pressure sensor sheet has enabled measurements to be obtained in a large number of subjects and the collection of clinical data in various diseases that present with symptoms of dysphagia.

Few investigations have reported the effects of applying sensors. Hind et al. (Hind et al. 2005) compared swallowing behavior using VF with and without the air-filled bulb under various conditions. They reported that the only statistically significant influences attributable to the presence of the pressure sensors were slight increases in residue in the oral cavity and UES, more frequent trace penetration of the laryngeal vestibule with effortful swallowing, and variances in oral clearance duration. In this study, we confirmed nonsignificant differences with and without the sensor sheet, thus suggesting that attachment of the sensor sheet did not affect swallowing behavior. Differences in materials and three-dimensional construction probably led to these different conclusions. Validation of these results may require EMG analysis, but this sensor sheet does not appear to affect the movement of hyoid and bolus.

Most research measuring tongue pressure only focuses on duration, maximal amplitude, and integral area. The authors had previously analyzed the sequence of tongue pressure generation and had reported that tongue pressure was initially generated in the anterio-median region, followed by the circumferential region, and the posterio-median region (Hori et al. 2009; Tamine et al. 2010; Hori et al. 2011). Furthermore, tongue pressure peaked quickly, and then decreased gradually before disappearing almost simultaneously at each measured part of the hard palate. In these reports, monophasic and biphasic waveforms of tongue pressure were observed. Despite these observations, the differences in the two patterns of tongue pressure have not been discussed to date.

The time sequences of the two patterns were compared in this study. We found that the temporal position of the monophasic pattern agreed with the posterior part of the biphasic pattern (Fig. 4). Furthermore, the maximum amplitude of tongue pressure with the monophasic pattern was almost the same as the second peak of the biphasic pattern (Fig. 5). Overall, we speculated that the monophasic tongue pressure and second wave of biphasic pattern might have no significant functional difference.

The wave of the monophasic pattern and the second wave of the biphasic pattern (especially at the Chs 1, 4, and 5) were located temporally at period from the tip of bolus reaches fauces to the tail of bolus passes UES. It suggested that the monophasic wave and second wave of biphasic pattern had temporal relationship with pharyngeal swallowing. On the other hand, the first wave of biphasic pattern at Chs 1, 4, and 5 existed at the period from the tip of tongue touches palate to tail of bolus passes fauces. The onset of biphasic pattern at the Chs 2 and 3 placed at the tip of bolus reaches fauces. It suggested that the anterior and circumference regions of tongue corresponded to Chs 1, 4, and 5 might seal bolus and the time differences between onset of Chs 1, 4, 5 and Chs 2, 3 might play an important role in propelling the bolus, as the tongue holds the bolus then propels it. These findings seem to be in agreement with the results observed in sagittal and coronal sections taken using ultrasonic diagnosis equipment (Stone and Shawker 1986; Hamlet et al. 1988) and VF (Taniguchi et al. 2008). This might explain why the biphasic pattern was observed frequently at Ch 1, 4, and 5, and it is reasonable that the mid-posterior region (Ch 3) would typically show a monophasic pattern.

The duration of tongue pressure in the biphasic pattern was longer than that in the monophasic pattern in this study. In our previous study, a longer duration was observed at Chs 4 and 5 during small volume swallowing. Based on these observations, the propelling movement is necessary, in small volume swallowing, as the tongue has to collect the bolus. The simultaneous measurement of tongue pressure and VF could lead these conclusions which may clarify the essential role of tongue pressure.

Some trials for the synchronous measurement of tongue movement with other equipment have been performed. Simultaneous examination with tongue pressure and supra-hyoid muscle activity reported that the onset of EMG was earlier than onset of tongue pressure and supra-hyoid muscle activity continued until the offset of tongue pressure (Taniguchi et al. 2008; Ono et al. 2009). Stone and Shawker (1986) observed the tongue and hyoid movement using real-time ultrasound and they reported that maximum tongue height was achieved 0.4 sec after the onset of hyoid movement and 0.1 sec prior to the arrival of the most anterior hyoid position. Moreover, they reported the beginning of tongue descent lagged from the onset of hyoid descent by an average of 0.2 sec. And, the hyoid is connected to tongue via the supra-hyoid muscles anatomically (Sawczuk and Mosier 2001). On the other hand, Steele et al. (2012) reported that they could not find the temporal relationship of tongue pressure and hyoid movement using real-time ultrasound and air-filled bulbs.

In this study, a certain temporal relationship was confirmed. That is, the onset of tongue pressure was later than the start of hyoid movement, and offset was observed between the hyoid at the up-forward position and hyoid repositioning. These results agree with a previous study (Stone and Shawker 1986). Furthermore, the offset of tongue pressure at Chs 4 and 5 showed a correlation with the arrival of the most up-forward hyoid position. The correlation formula indicates that the time lag from arrival of the most up-forward hyoid position to the offset of tongue pressure is 0.14–0.23 sec. This suggests that tongue pressure generation has a time-oriented relationship with the hyoid elevation.

In this study, we used VF, which is able to observe hard tissue and bolus, in order to analyze the movement of hyoid and bolus. As the time resolution of VF is only 30 Hz, it may have limitations with regard to detailed analysis.

From the tongue pressure measured in this study, we can discuss only the state of tongue-palate contact. The kinetics of tongue body such as slipping or rolling could not be caught. Although the significance of the magnitude of tongue pressure has not been analyzed, Palmer et al. (2008) reported that tongue pressure has a relationship with supra-hyoid muscle activity. The magnitude or slope of tongue pressure may have relationship with the transport of bolus or hyoid kinetics. Further analysis to assess the magnitude or slope of tongue pressure is, therefore, necessary.

Although liquid was used as a bolus in this study, if the relationship between tongue pressure and hyoid movement is recognized in the case of other textures, the role of tongue pressure would be clarified in greater detail. Previous studies demonstrated that the bolus transit time was affected by the initial bolus conditions including its texture, volume, consistency during voluntary swallowing (Taniguchi et al. 2008). Furthermore, it was also found that the number of swallows and tongue pressure were much less during liquid swallowing than jelly swallowing (Hayashi et al. 2013). However, we only chose thin liquid from the viewpoint of the subject's strain and radiation exposure. In this regard, effects of bolus consistency on the tongue movements or lingual muscle activity in the oral stage of swallowing should be investigated with other than liquid.

Despite these limitations, tongue pressure has a temporal relationship with hyoid movement. Konaka et al. (2010) who assessed tongue pressure in stroke patients mentioned that the more than two peaks were observed in dysphagic patients. The presently observed waveform and temporal coordinated sequence of tongue pressure could thus be applied to assessment of tongue movement dysfunction or disharmonic bolus movement. Furthermore, it may help to devise effective tongue training methods for improving tongue behavior during swallowing.

## Conclusions

The present results suggested the temporal coordination among tongue pressure generation, hyoid and bolus movement, and muscle activity. That is, the tongue pressure was produced for bolus propulsion, and was closely related to hyoid movement temporally during swallowing. The findings we clarified in this study can be useful for clinical work of dysphasia rehabilitation.
